# Study on the Influence of Diversity and Quality in Entropy Based Collaborative Clustering

**DOI:** 10.3390/e21100951

**Published:** 2019-09-28

**Authors:** Jérémie Sublime, Guénaël Cabanes, Basarab Matei

**Affiliations:** 1 ISEP, DaSSIP Team–LISITE, 10 rue de Vanves, 92130 Issy-Les-Moulineaux, France; 2 University Paris 13, Sorbonne Paris Cité, LIPN-CNRS UMR 7030, 99 av. J-B Clément, 93430 Villetaneuse, France; guenael.cabanes@lipn.univ-paris13.fr (G.C.); matei@lipn.univ-paris13.fr (B.M.)

**Keywords:** collaborative clustering, clustering quality, entropy, diversity

## Abstract

The aim of collaborative clustering is to enhance the performances of clustering algorithms by enabling them to work together and exchange their information to tackle difficult data sets. The fundamental concept of collaboration is that clustering algorithms operate locally but collaborate by exchanging information about the local structures found by each algorithm. This kind of collaborative learning can be beneficial to a wide number of tasks including multi-view clustering, clustering of distributed data with privacy constraints, multi-expert clustering and multi-scale analysis. Within this context, the main difficulty of collaborative clustering is to determine how to weight the influence of the different clustering methods with the goal of maximizing the final results and minimizing the risk of negative collaborations—where the results are worse after collaboration than before. In this paper, we study how the quality and diversity of the different collaborators, but also the stability of the partitions can influence the final results. We propose both a theoretical analysis based on mathematical optimization, and a second study based on empirical results. Our findings show that on the one hand, in the absence of a clear criterion to optimize, a low diversity pool of solution with a high stability are the best option to ensure good performances. And on the other hand, if there is a known criterion to maximize, it is best to rely on a higher diversity pool of solution with a high quality on the said criterion. While our approach focuses on entropy based collaborative clustering, we believe that most of our results could be extended to other collaborative algorithms.

## 1. Introduction

Collaborative clustering is a relatively new discipline with few existing works available in the literature [1,2,3,4,5,6,7]. Its main goal is to reveal the common underlying structures found by different unsupervised algorithms while analyzing data that may be split on different sites. The main interest of collaborative clustering is to help local clustering algorithms to either confirm or improve their respective models and clustering results by using information provided by other algorithms and that would be otherwise unavailable to them. This type of algorithm has proved very useful to perform clustering techniques on data spread in multiple sites under different representations while considering privacy issues [8].

In a sense it is the unsupervised equivalent of Ensemble Learning methods that have been around for a while in supervised Learning [9,10,11,12]. As such, collaborative clustering is a difficult problem that combines several of the major issues from both clustering and ensemble learning: Choosing the right number of clusters, assessing the quality of clustering results, and weighting the influence of the different collaborating algorithms. Both ensemble learning methods and collaborative methods are generally two-step frameworks: (1) The algorithms operate locally and build their own models; (2) The algorithms work together by sharing their information and results. However the similarities between collaborative cluster and ensemble learning end here, and both domains have major differences. The first major difference is that ensemble learning methods usually aim for a unique consensus result, while collaborative learning methods aim for a mutual improvement of all the results. Furthermore, ensemble learning methods can rely on a ground-truth to assess the quality of each result while collaborative learning cannot because it is unsupervised. As a consequence, collaborative clustering methods suffer from a higher risk of negative collaboration caused by the participation of a weak algorithm in the collaborative process.

In this paper, we are interested in studying what makes a collaboration efficient and trying to predict the outcome in advance. To do so, we focus on the *Entropy Based Collaborative Clustering* algorithm (EBCC) [13], a method developed recently and that allows for collaboration between different types of clustering algorithms that are probabilistic, something that is not possible with most other collaborative methods which are mono-algorithm. Our proposed analysis combines two elements:

- An optimization based method whose aim is to find the best weighting between algorithms in order to decide with which to collaborate or not. This is achieved using Karush Kuhn Tucker (KKT) optimization and the result gives an insight on the importance of diversity to achieve positive collaborations. This first contribution is an extension of two previous conference papers [14,15].
- An empirical simulation, in which we run multiple instances of the EBCC algorithm with solutions of various quality and diversity in order to assess the influence of these two parameters. From the results, we use linear regression analysis to propose a predictive model for the outcome of collaborative clustering.

The remainder of this paper is organized as follows: Section 2 introduces some related works on collaborative clustering as well as other studies similar to this work. Section 3 presents the Entropy Based Collaborative algorithm as well as useful notations for the rest of the paper. Section 4 features the mathematical analysis of the optimal weights to get the best possible results from a mathematical point of view using KKT optimization. Section 5 is the empirical study contribution of this paper which assesses the actual outcome of collaborations between solutions of various quality and diversity and tries to build a model based on the results. Finally, this paper ends with a conclusion and perspectives on future works.

## 2. Related Works

The issue of result quality when dealing with multiple algorithms has been an ongoing challenge even before collaborative clustering. The fields of supervised ensemble learning [16], unsupervised ensemble learning and multi-view clustering [17,18] have indeed the same problematic as the one we tackle in this paper for collaborative clustering: the issue of multiple truths [19], quality of the view and diversity of the ensembles has been widely studied in the literature.

Collaborative clustering suffers from the same difficulties of dealing with several algorithms providing multiple partitions of unknown quality and with a diversity of result that can also vary. Furthermore, when dealing with the same data under multiple representation, a good quality partition in a given view may prove to be of a very low quality in another view when multiple truths exist and if the said views hold incompatible structures. This was first studied for collaborative clustering applied to semantic web analysis [20] where the authors try to reconcile different facets semantic web analysis using collaborative fuzzy C-Means.

More recently, other studies [21,22,23] similar to Section 4 of this work have been proposed to optimized the weights between local and collaborative terms, as well as the inter-collaboration weights between algorithms. These studies, while limited to approaches of collaborative clustering based on Self-Organizing Map and Generative Topographic Mapping [2,24,25], use gradient descent optimization to optimize the weights as the algorithm runs. The main difference between these studies and our contributions are the following:

1. What we propose in this paper is not limited to a single algorithm or a single family of algorithm. As we explain in Section 4, our optimization model of the weight is generic and can apply to most collaborative clustering algorithms.
2. The optimization model for the weights in this paper does not rely on derivation and gradient descent but on KKT optimization. It is therefore very generic.
3. We provide an interpretation of the optimal weights found by our proposed method and compare them with the empirical results to see if theory and practice agree or not.

In [26], the authors propose a recent study which focuses on the influence of quality and diversity for the partitions involved in collaborative clustering based on the SOM algorithm [24]. This work is based mostly on empirical results and uses the same point clouds technique as in Section 5 of our paper. Due to these similarities, and while it deals with a collaborative algorithm different from the one in this work, we use this study for comparison purposes with our own results.

## 3. Entropy Based Collaborative Clustering

In this section, we remind the principle of the EBCC algorithm as introduced in [13]. EBCC deals with horizontal collaboration, meaning several clustering algorithms processing the same objects represented by different features in different views, like in multi-view clustering [19].

### 3.1. Notations

Given *J* views, let us consider $A=\{A^{1},\cdots ,A^{J}\}$ a set of *J* clustering algorithm.

Each algorithm $A^{i}=\left\{X^{i},S^{i},\Theta^{i},K_{i}\right\}$ searches for $K_{i}$ clusters with a distribution of parameters $\Theta^{i}$ in a subset $X^{i}=\left\{x_{1}^{i},\cdots ,x_{N}^{i}\right\},x_{n}^{i}\in R^{d_{i}}$ of real valued data. This algorithm will then provide a local partition $S^{i}={\left(s_{n,c}^{i}\right)}_{K_{i}\times K_{i}}$ in the form of a vector which gives the probability of a data $x_{n}^{i}$ to belong to a given cluster *c*, $c\in [1..K_{i}]$.

While it is never accessible by a single algorithm, the full data with all view are denoted by $X=\{X^{1},\cdots ,X^{J}\}$. In a similar way, we define $\Theta =\{\Theta^{1},\cdots ,\Theta^{J}\}$ the set of all parameters, and $S=\{S^{1},\cdots ,S^{J}\}$ the set of all local solutions.

We also note $Z^{i}:\Omega \to \left[1..K_{i}\right]$ the latent random vector linked to the solutions of algorithm $A^{i}$, and as such $P(Z^{i}|X^{i},\Theta^{i})$ is the a posteriori distribution of $Z^{i}$ conditionally to $X^{i}$ and $\Theta^{i}$.

Let $w_{ab}^{ij}$ be the percentage of data associated to cluster $a\in [1..K_{i}]$ by $A^{i}$ that belong to cluster $b\in [1..K_{j}]$ for algorithm $A^{j}$: $w_{ab}^{ij}=P\left(Z_{n}^{j}=b|Z_{n}^{i}=a\right)$. As such each matrix $W^{ij}={\left(w_{ab}^{ij}\right)}_{\left(K_{i}\times K_{j}\right)}$ is the confusion matrix for the clusters of solutions $S^{i}$ and $S^{j}$.

Finally, in Equation (1), let us define H the global entropy of the collaborative system for all algorithms. This measure based on the probabilistic confusion entropy [27] is used as an entropy measure between the different solutions and as a stopping criterion for the EBCC algorithm.

(1) $$H=\sum_{i=1}^{J}\sum_{j\neq i}^{J}\frac{-1}{K_{i}\times \log\left(K_{j}\right)}\sum_{l=1}^{K_{i}}\sum_{m=1}^{K_{j}}w_{lm}^{ij}\times \log\left(w_{lm}^{ij}\right)$$

### 3.2. Algorithm

The canonical form of the global fitness function for the EBCC algorithm is defined by using the global log-likelihood of the *J* algorithms, when local and collaborative terms have the same weight this writes as follows:

(2) $$L_{g}\left(S,\Theta \right)=L\left(S,\Theta \right)+C\left(S\right)$$

with L(S,Θ) the local term linked to the log-likelihood of the partition local algorithm $A^{i}$ and C(S) is a collaborative term assessing the log-likelihood of having each local partition, knowing the other partitions and models. In the original paper for the EBCC [13], the authors make the argument that while le local term can be developed using the independence of the models, this is not the case for the collaborative term which ends up being non-tractable and impossible to optimize.

To alleviate this problem, the authors proposed an alternative objective function $\tilde{L}\left(S,\Theta \right)$ which is a minimizer of L(S,Θ). As such, maximizing this alternate function has the result of maximizing the original objective function. This function $\tilde{L}\left(S,\Theta \right)$ keeps the original local term and use *combination functions* that estimate the likelihood of a data $x_{n}^{i}$ to be in a given cluster locally, knowing the clusters inside which it is located in the other views, and the likelihood of these clusters to be correct. This is done using the confusion matrices $W^{ij}$ with the most likely combinations to reduce the computational cost. After several transformations and simplifications, the EBCC algorithm ends up with the objective function $\tilde{L}\left(S,\Theta \right)$ shown in Equation (3) where the $\beta_{ji}$ are the weights determining the strength of the collaboration from algorithm $A^{j}$ to algorithm $A^{i}$.

(3) $$\tilde{L}\left(S,\Theta \right)=\sum_{i=1}^{J}\left(P\left(Z^{i}|X^{i},\Theta^{i}\right)+\frac{1}{J-1}\sum_{j\neq i}\frac{\beta_{ji}}{N}\sum_{n=1}^{N}w_{s_{n}^{j},s_{n}^{i}}^{ji}\right)$$

This simplification is obtained by starting from the canonical form given in [13] and [14] which shows a datawise objective function during the update phase:

(4) $$P\left(Z_{n}^{i}|X_{n}^{i},\Theta^{i},S\right)=\left(1-\lambda \right)\cdot P\left(Z_{n}^{i}|X_{n}^{i},\Theta^{i}\right)+\lambda \cdot P\left(Z_{n}^{i}|Z_{n}\setminus Z_{n}^{i}\right)$$

From Equation (4), Equation (3) is obtained by summing over all the data and all the views to have the global objective function, and by using the additive combination function described in the same works. The confusion matrix $W^{ij}$ is used here as an a posteriori approximation of the probability of having $x_{n}$ belonging to a certain cluster in view *i* knowing the cluster is belongs to in view *j*. Please note that the original paper [13] proposes several alternative objective functions based on the confusion matrix $W^{ij}$. Some of them are in product form, and other in sum form and introduce the weights collaboration $\beta_{ji}$ as a refinement. Furthermore, the form presented in Equation (3) is based on the most commonly used sum form and assumes an equal weight given to local and collaborative term, hence the $\frac{1}{J-1}$ for normalization purposes in the collaborative term.

From there, the EBCC algorithm optimizes (3) as follows:

1. **Local step:** Initialization of all $S^{i}$ and $\Theta^{i}$ using the local algorithms to maximize all $P(Z^{i}|X^{i},\Theta^{i})$.
2. **Collaborative step:** Repeat the following alternate maximization steps until the global system entropy H from Equation (1) stops improving:
  - Find *S* that maximizes $\tilde{L}\left(S,\Theta \right)$ for Θ fixed, update *S*.
  - Find Θ that maximizes $\tilde{L}\left(S,\Theta \right)$ for *S* fixed, update Θ.

The original EBCC paper shows that $\tilde{L}\left(S,\Theta \right)\leq L_{g}\left(S,\Theta \right)$ and that maximizing $\tilde{L}\left(S,\Theta \right)$ also directly optimize the global entropy of the system. Therefore, since we are not able to maximize $L_{g}\left(S,\Theta \right)$, we maximize $\tilde{L}\left(S,\Theta \right)$ instead. As such, the variables (S,Θ) maximizing $\tilde{L}\left(S,\Theta \right)$ will also maximize $L_{g}\left(S,\Theta \right)$.

## 4. Mathematical Study on the Influence of Diversity

In this section we study the optimal confidence weights to give to the different algorithms during a collaboration process. Please note that while our paper focuses on the EBCC algorithm [13], the results of this section can be extended to almost any collaborative clustering algorithm, so long as their objective function can take either of the complementary forms given in Equations (5) and (6) below, where $L(X^{i},S^{i},\Theta^{i})$ is the local term, the $\beta_{ji}$ are weights to be determined that give the strength of the collaboration from $A^{j}$ to $A^{i}$, $\Delta (\left[S^{i},\Theta^{i}\right],\left[S^{j},\Theta^{j}\right])$ the collaborative term in the form of a divergence or distance function, and $C(\left[S^{i},\Theta^{i}\right],\left[S^{j},\Theta^{j}\right])$ the collaborative term in the form of a consensus or similarity function.

(5) $$L^{d}\left(S,\Theta \right)=\sum_{i=1}^{J}\left(L\left(X^{i},S^{i},\Theta^{i}\right)-\sum_{j\neq i}\beta_{ji}\cdot \Delta \left(\left[S^{i},\Theta^{i}\right],\left[S^{j},\Theta^{j}\right]\right)\right)$$

(6) $$L^{c}\left(S,\Theta \right)=\sum_{i=1}^{J}\left(L\left(X^{i},S^{i},\Theta^{i}\right)+\sum_{j\neq i}\beta_{ji}\cdot C\left(\left[S^{i},\Theta^{i}\right],\left[S^{j},\Theta^{j}\right]\right)\right)$$

When looking at the literature, most existing collaborative approaches fit either of these two forms: the original collaborative fuzzy C-Means algorithm [28], Kohonen based approaches to collaborative clustering [2,24], GTM based approaches [25], and also information theory based approaches to collaborative clustering [6]. As one can see, this is very generic.

For the purpose of simplicity and since divergence and consensus form of collaborative terms are complementary, we will focus on the consensus form from Equation (6). We simplify it into Equation (7) to make the notations lighter, with $L_{i}=L\left(X^{i},S^{i},\Theta^{i}\right)$ and $C_{ij}=C\left(\left[S^{i},\Theta^{i}\right],\left[S^{j},\Theta^{j}\right]\right)$.

(7) $$L=\sum_{i=1}^{J}\left(L_{i}+\sum_{j\neq i}\beta_{ji}\cdot C_{ij}\right)$$

### 4.1. KKT Optimization Model

The method that we propose consists in optimizing Equation (6) under the Karush-Kuhn-Tucker conditions (KKT) [29], with the goal of finding the ideal $\beta_{ji}$. In a second step, we will interpret the expression found with the aforementioned method.

First, we can see that by construction the $\beta_{ji}$ have no influence on the local term, so we can focus only on the collaborative one. From there, finding the $\beta_{ji}$ that maximize the collaboration outcome under the constraint $\sum_{j\neq i}^{J}\beta_{ji}=1$ gives us the following system:

(8) $$\left\{\begin{array}{c} B={\mathrm{argmax}}_{B}\sum_{i=1}^{J}\sum_{j\neq i}\beta_{ji}C_{ij}\\ \forall i\;\sum_{j\neq i}^{J}\beta_{ji}=1\\ \forall \left(i,j\right)\;\beta_{ji}\geq 0 \end{array}\right.$$

From this system, by using the Lagrange multipliers ν and λ, we get the following KKT conditions:

(9) $$\forall \left(i,j\right),i\neq j\left\{\begin{array}{c} \left(a\right)\;\beta_{ji}\geq 0\\ \left(b\right)\;\sum_{j\neq i}^{J}\beta_{ji}=1\\ \left(c\right)\;\lambda_{ji}\geq 0\\ \left(d\right)\;\beta_{ji}\cdot \lambda_{ji}=0\\ \left(e\right)\;C_{ij}-\lambda_{ji}-\nu_{i}=0 \end{array}\right.$$

With (c) and (e), we have:

(10) $$\lambda_{ji}=C_{ij}-\nu_{i}\geq 0$$

Let us suppose that there is a *k* so that $\beta_{ki}>0$. Then with (d), we have: $\lambda_{ki}=0$. And with Equation (10), we have: $\nu_{i}=C_{ik}$. Then, using this information we can say that:

(11) $$\forall j\neq k\left\{\begin{array}{c} \beta_{ji}\neq 0\Rightarrow C_{ij}=C_{ik}\Rightarrow \lambda_{ji}=0\\ \beta_{ji}=0\Rightarrow \lambda_{ji}=C_{ij}-C_{ik}\geq 0 \end{array}\right.$$

From the second line of Equation (11), we can conclude the following:

(12) $$\beta_{ji}\neq 0\Rightarrow C_{ij}=\max_{k}C_{ik}$$

Then, if we use (d) and (e), we have:

(13) $$\beta_{ji}\left(C_{ij}-\nu_{i}\right)=0$$

If we sum Equation (13) over *j* and use (b), we have:

(14) $$\nu_{i}=\sum_{j\neq i}^{J}\beta_{ji}\cdot C_{ij}$$

For Equation (14) to be correct while respecting the constraints given in Equations (11) and (12), the only solution is:

(15) $$\forall j\neq i,\beta_{ji}^{0}=\left\{\begin{array}{cc} \frac{1}{Card(C_{ij}=max_{k}\left(C_{ik}\right))} & \mathrm{if}\;C_{ij}=max_{k}\left(C_{ik}\right)\\ 0 & \mathrm{otherwise} \end{array}\right.$$

It is possible to get a relaxed version of this result by modifying the system from Equation (8) as follows:

(16) $$\left\{\begin{array}{c} B={\mathrm{argmax}}_{B}\sum_{i=1}^{J}\sum_{j\neq i}\beta_{ji}\cdot C_{ij}\\ \forall i\;\sum_{j\neq i}^{J}{\left(\beta_{ji}\right)}^{p}=1,\;p\in N^{*},\;p>1\\ \forall \left(i,j\right)\;\beta_{ji}\geq 0 \end{array}\right.$$

It is worth noting that the $\beta_{ji}$ of this system are not the same ones as in Equation (9). For the sake of completeness we will compute them and show that they can still be used in the original equation. With this system, we get the following new KKT conditions:

(17) $$\forall \left(i,j\right),i\neq j\left\{\begin{array}{c} \left(a\right)\;\beta_{ji}\geq 0\\ \left(b\right)\;\sum_{j\neq i}^{J}{\left(\beta_{ji}\right)}^{p}=1,\;p\in R^{+*}\\ \left(c\right)\;\lambda_{ji}\geq 0\\ \left(d\right)\;\beta_{ji}\cdot \lambda_{ji}=0\\ \left(e\right)\;C_{ij}-\lambda_{ji}-\nu_{i}\cdot \left(p\cdot {\left(\beta_{ji}\right)}^{p-1}\right)=0 \end{array}\right.$$

If we consider the case $\beta_{ji}\neq 0$ and $\lambda_{ji}=0$ in Equation (d), then with (e) we have:

(18) $$\beta_{ji}={\left(\frac{C_{ij}}{p\cdot \nu_{i}}\right)}^{\frac{1}{p-1}}$$

From Equation (18) and (b), we have:

(19) $$1={\left(p\cdot \nu_{i}\right)}^{\frac{-p}{p-1}}\sum_{j\neq i}{\left(C_{ij}\right)}^{\frac{p}{p-1}}={\left(\nu_{i}\right)}^{\frac{-p}{p-1}}\sum_{j\neq i}{\left(\frac{C_{ij}}{p}\right)}^{\frac{p}{p-1}}$$

Then we can write:

(20) $$\nu_{i}=\frac{1}{p}{\left(\sum_{j\neq i}{\left(C_{ij}\right)}^{\frac{p}{p-1}}\right)}^{\frac{p-1}{p}}$$

Then by injecting the expression of $\nu_{i}$ into Equation (18), ∀(i,j),i≠j,p>1 we have:

(21) $$\begin{array}{cc} \beta_{ji}^{+} & =\frac{{\left(C_{ij}\right)}^{\frac{1}{p-1}}}{{\left(\sum_{k\neq i}^{J}{\left(C_{ik}\right)}^{\frac{p}{p-1}}\right)}^{\frac{1}{p}}} \end{array}$$

The main inconvenient of this second result is that it requires the extra parameter *p* too be fixed before running the collaborative clustering method. Since some collaborative methods already have a lot of parameter to be decided, following a method proposed in [30], where the authors faced a similar optimization problem for multi-view clustering, it is possible to propose a third parameter free system using a product constraint instead of a sum:

(22) $$\left\{\begin{array}{c} B={\mathrm{argmax}}_{B}\sum_{i=1}^{J}\sum_{j\neq i}\beta_{ji}\cdot C_{ij}\\ \forall i\;\prod_{j\neq i}^{J}\beta_{ji}=1,\\ \forall \left(i,j\right)\;\beta_{ji}\geq 0 \end{array}\right.$$

This problem can be solved using a logarithm form to transform the product into a sum, and under the extension of the KKT conditions to invex functions [31]. After developing the system as we did above, we get the following result:

(23) $$\begin{array}{cc} \beta_{ji}^{*} & =\frac{C_{ij}}{{\left(\prod_{k\neq i}C_{ik}\right)}^{\frac{1}{J-1}}} \end{array}$$

### 4.2. Results Interpretation

We begin by analyzing the first result from Equation (15) which basically says that in the most constrained case, each view of a collaborative framework should only collaborate and acquire information from the view that has the closest model and should not collaborate at all with the other views. The model specifies that in the case where several views have equally close models, information should be acquired from all of them with an equal exchange link. We can reasonably say that mostly pairwise exchanges between most similar views is very restricting, hence our proposal of a relaxed version of the weights in Equation (16).

In Equation (21), we have a second result which still highlights that collaboration should primarily occur with views that have similar partitions, with more or less flexibility depending on the value of *p*. Based on the value of *p* the information coming from divergent models should be given some importance too based on their degree of divergence. In fact, we can see that when *p* grows towards infinity all weights become equal.

The two results in Equations (15) and (21) are a continuity as both encourage to different degrees collaboration with algorithms that have similar partition, and thus a low diversity. When analyzing the expression of the parameter free $\beta_{ji}^{*}$ from Equation (23), we can see that the interpretation is the same: the more similar the partitions, the stronger the collaboration weights. As such, we clearly see that on all 3 cases, the results seem to indicate that better results happen when solutions with a low diversity collaborate.

With this in mind, we can conclude that the optimization process used to find the partitions and in fine the model would have two interesting properties:

1. Optimal collaborative clustering aims at reducing the divergences between similar partitions.
2. Optimal collaborative clustering does not encourage diversity and reduces the exchanges between partitions or models that are too dissimilar.

The first property makes sense, since most collaborative models as well as EBCC aim at reducing the entropy between all solutions, and thus lower the diversity. As such, favoring collaboration between high similarity algorithms makes sense. This result is also is interesting because it relates with the concept of stability in clustering partitions [32,33]. Indeed, by encouraging views with similar results to work together in an unsupervised context, it increases the chances of achieving better results, since structures found in several views can be considered stable which is a good and unbiased quality index for exploratory data mining.

The second and first properties together have direct applications to tackle the problem of noisy view detection. If we consider multi-view data where the same objects are represented using different redundant views, we know that the two main problems are: views that contain mostly noise, views or groups of views that contains different and incompatibles data structures. Using any of our proposed weighting systems, the noisy views would not hinder the others by sending wrong information because they are too different and will have low exchange links toward every other views. As for groups of views containing different structures, our weighting systems would mostly foster exchanges between similar views, thus creating meta-clusters of similar views. These meta-clusters of views would still exchange information through the views that are compatible with several meta-clusters, and noisy views would remain isolated as outliers.

Using the graph of the weights $\beta_{ji}$, it would be very simple to detect views containing mostly noisy features, but also to remove redundant attributes by detecting communities of hyper-connected views forming clusters in the graph and removing some of them.

### 4.3. Datasets and Indexes

In this section as well as in Section 5, we use the following dataset:

- This Iris Dataset (UCI): This data set has 150 instances of iris flowers described by 4 integer attributes. The flowers can be classified in 3 categories: Iris Setosa, Iris Versicolour and Iris Virginica. Class structures are well behaved and the class instances are balanced (50/50/50). For the purpose of this experiment, we create artificial view containing only 3 of the 4 attributes.
- The Wine Dataset (UCI): This data set contains 178 instances of Italian wines from 3 different cultivars. All wines are described by 13 numerical attributes and the classes to be found are the 3 cultivars of origin. Class structures are well behaved in this data set, but the class instances are unbalanced (59/71/48).
- The EColi Dataset (UCI): This data set contains 336 instances describing cells measures of Escherichia Coli bacteria. The original data set contains 7 numerical attributes (we removed the first attribute containing the sequence name). The goal of this data set is to predict the localization site of proteins by employing some measures about the cells. There are 4 main site locations that can be divided into 8 hierarchical classes.
- The Wisconsin Data Breast Cancer (UCI): This dataset has 569 instances with 30 variables from 3 different cells that can easily be split into 3 natural views of 10 attributes. Each data observation is labeled as benign (357) or malignant (212).
- The Image Segmentation dataset (UCI): The 2310 instances of this data set were drawn randomly from a database of 7 outdoor images. The images were hand segmented to create a cation for every pixel. Each instance is a 3 × 3 region represented by 19 attributes and there are 7 classes to be found. The attributes can be split into views based on the colors (RGB).
- The VHR Strasbourg dataset ([34,35]): It contains the description of 187.058 segments extracted from a very high resolution satellite image of the French city of Strasbourg. Each segment is described by 27 attributes that can be split between radiometical attributes, shape attributes, and texture attributes. The data set is provided with a partial hybrid ground-truth containing 15 expert classes. Due to its large size, this dataset is not used in all our experiments.

For our studies, we consider two internal quality indexes: the Davies-Bouldin index [36] and the Silhouette index [37]. The Davies-Bouldin index is a strictly positive non-normalized index that is better when it is smaller. It is generally associated with the K-Means algorithm for which it was originally design as a quality measure. The Silhouette index is another index common to assess clustering results. It can be applied to specific data, isolated cluster or a full partition. The Silhouette index is normalized between −1 and 1: a positive value close to 1 indicates a good partition, well formed cluster, or that a data is in the right cluster. On the other hand, negative values for the Silhouette index indicate wrong allocations or ill-formed clusters.

### 4.4. Results with the Optimized Weights under KKT Conditions

In our experimental protocol, for each data set, we created 5 subsets by removing some of the attributes randomly. Each subset was initialized using the EM algorithm for the Gaussian mixture model, and then optimized using the EBCC algorithm. We then ran our collaborative framework with the weighted sum constraint using with the parameter p=2.

The results of this experiment are shown in Table 1 where the average result over a dozen simulations are shown in the main cells, as well as the best and worst results over all simulations that are shown between brackets.

As one can see, our proposed framework has overall positive results for two different clustering indexes. While it is true that the improvement after collaboration is not huge, our proposed weighting method has the advantage of resulting in few cases of negative collaborations (results getting worst after collaboration). These results highlight that our weighting system has the positive aspect of reducing the number of cases of negative collaborations, but also the flaw that it may seems to lead to only minor improvements of the original solutions after collaboration.

## 5. Empirical Study on the Influence of Diversity and Quality

With the mathematical model from Section 4 showing that optimal collaborative clustering should have models with a low diversity, in this section we propose an experimental study to verify this result and test other factors. Our goal is to test if theory and practice agree. To do so, we apply the EBCC algorithm to several datasets, and we test how model diversity and quality indexes may influence the results in practice.

### 5.1. Experimental Protocol

Our methodology for this second study is the following: we use the original version of the EBCC algorithm as it was introduced in Section 3, without the KKT weighting system from the previous section. We first evaluate the performances of our collaborative framework depending on the initial quality and diversity of the solutions. Then, we use point clouds to display the results of a large number of simulations over several data sets. From there we make an analysis of the influence of quality indexes and diversity to predict the outcome of a collaboration. We also use this section to assess some extra properties of our collaborative framework. Then in Section 5.3, we use the point clouds to build a regression model to predict the outcome of a collaboration in term of internal quality criterion.

Unlike the previous one, this study considers the diversity between the partition that collaborate together as a parameter. Note that in the context of collaborative clustering, ensemble learning and multi-view clustering, the notion of diversity refers to the variety of clustering partitions present in the pool of views and algorithms. Many measures can be used for this [16], including rand index, disagreement measures, and entropies. And since we are using the EBCC algorithm that relies on a entropy measure as a stopping criterion to optimize, we use the local oriented entropy of the solutions that collaborate together as a diversity measure. This entropy between two solutions $S^{i}$ and $S^{j}$ is shown in Equation (24) and is directly derived from the global system entropy from Equation (1).

(24) $$H_{i,j}=\frac{-1}{K_{i}\times \ln\left(K_{j}\right)}\sum_{l=1}^{K_{i}}\sum_{m=1}^{K_{j}}w_{lm}^{ij}\ln\left(w_{lm}^{ij}\right)$$

Before starting with the experimental evaluation of our collaborative framework, we want to give some specific vocabulary and elements that will be useful to interpret the point clouds.

First of all, in the following sections we will evaluate the evolution of quality and diversity from the point of view of individual algorithms: If we consider *Q* a quality index (internal or external), then GQ the raw quality gain will be evaluated individually from the point of view of each algorithm. In short anything related to a quality index (*Q*, GQ, ΔQ) will be computed from the point of view of a *local algorithm* in the *local feature space*, while diversity measures will be computed based on the other *collaborating algorithms*.

Based on these definitions, earlier works have defined a collaboration to be a “positive collaboration” when GQ>0 and a “negative collaboration” otherwise.

**Figure 1:**
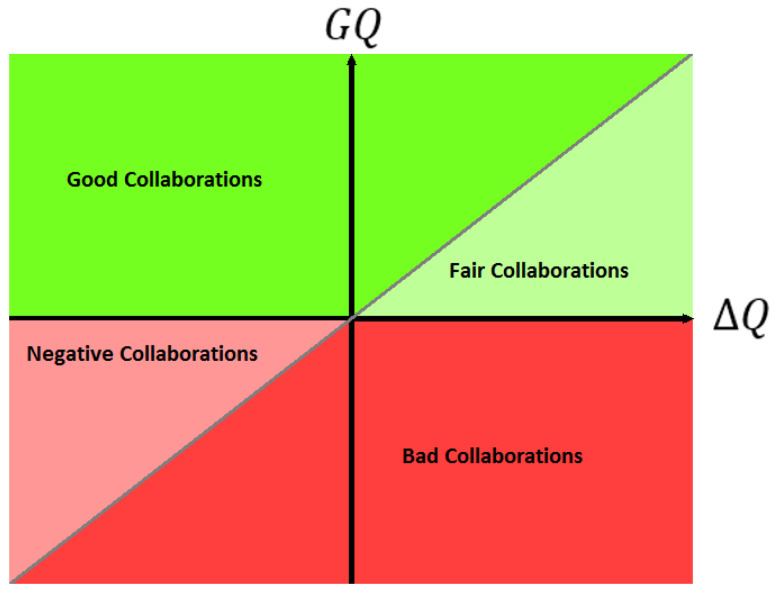
The different types of outcome for a collaboration.

If we consider ΔQ the average quality difference before collaboration between two given algorithms solutions in the feature space of the observed algorithm, Figure 1 shows what a typical GQ=f(ΔQ) point cloud diagram will look like. Points in the green area would be positive collaborations while points in the red area would be negative ones.

We extend this vocabulary as follows by adding some contrast to the possible outcomes of a collaboration:

- A collaboration outcome where the local result is improved so that it becomes better than the average results of the other collaborators (grey diagonal line) before collaboration will called a *good collaboration*.
- If the result improves but remain lower than than the average quality of the other collaborators before collaboration, it is a *fair collaboration*.
- If the result gets worse while remaining above the average quality before collaboration, then it is a *negative collaboration*.
- Finally, if the result are worse and are bellow the the average quality before collaboration, such collaboration is not just a negative collaboration, it is a *bad collaboration*. These terms are illustrated in the diagram shown in Figure 1.

Since we needed a large number of experiments to draw point cloud covering a large spectrum of quality and diversity between solutions, we needed to generate a large number of solutions to collaborate together. To do so, we used the following generation protocol: We used solutions generated by several randomly initialized GMM algorithms working on different subset of attributes as a basis. We also used the classification solutions for each dataset. These start solutions were then randomly modified with a more or less high mutation rate. We repeated the process twice to have 3 generations of solutions. Finally, we injected some completely randomly generated clustering partitions too the mix.

While the process used to generate a lot of simulations might be perfectible, we believe that it covered a fair amount of diversity and quality between solutions.

### 5.2. Point Clouds Visualization and Interpretation

The results are shown in the form of four point clouds for all data sets:

- The raw Silhouette index difference between the two collaborators depending on the initial diversity. These are not experimental results and are just here to show that our simulations covered most possible cases of differences in quality and diversity prior to the collaborative process.
- The Silhouette index raw improvement depending on the initial entropy between the collaborators.
- The Silhouette index raw improvement depending on the initial Silhouette index raw difference between the two collaborators.
- The Davies-Bouldin index raw improvement depending on the initial Davies-Bouldin index raw difference between the two collaborators.

**Figure 2:**
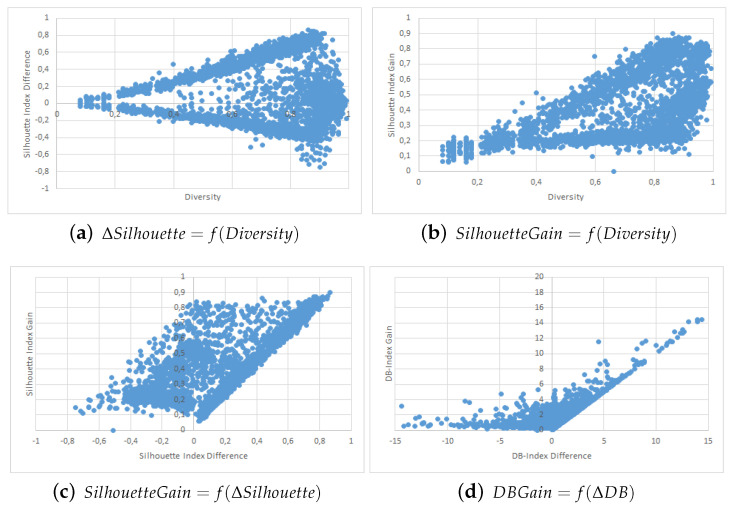
Iris Data Set Point Clouds.

**Figure 3:**
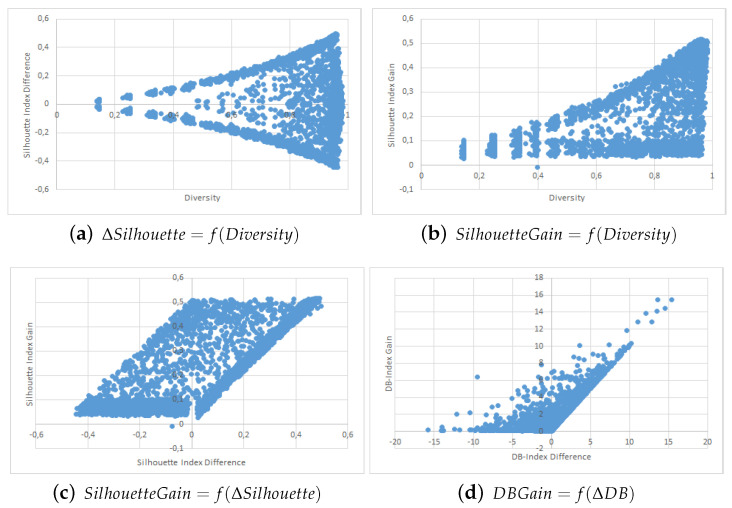
WDBC Data Set Point Clouds.

**Figure 4:**
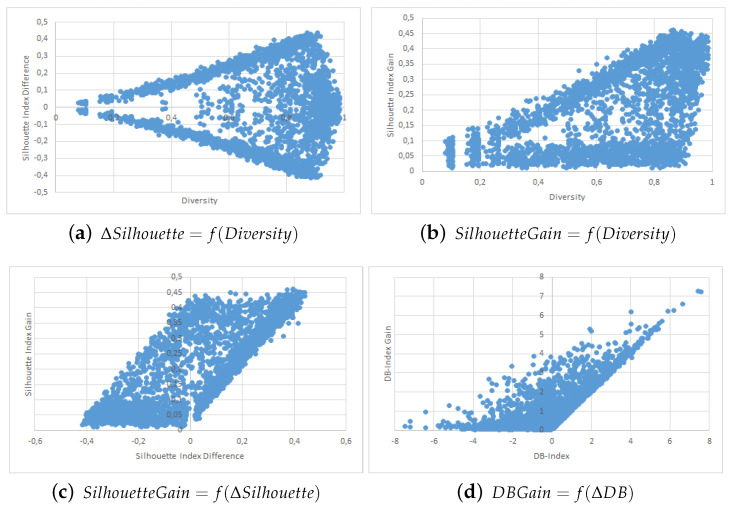
Wine Data Set Point Clouds.

**Figure 5:**
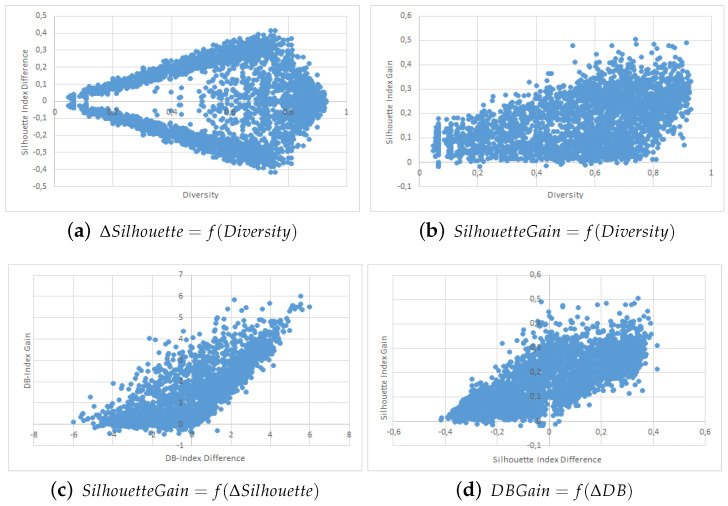
Image Segmentation Data Set Point Clouds.

**Figure 6:**
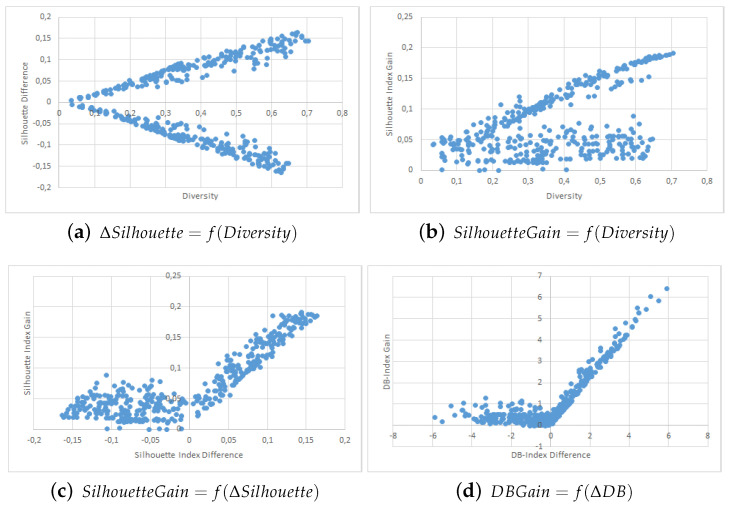
VHR Strasbourg Data Set Point Clouds.

We used point clouds containing 1500 simulated collaborations (3000 points since the collaboration is evaluated both ways) for the Iris, WDBC, Wine and Image data sets, and 250 simulated collaborations (500 points) for the VHR Strasbourg data set because of computation time issues. The point clouds for these experiments are shown in Figure 2, Figure 3, Figure 4, Figure 5 and Figure 6.

We first want to analyze Figure 2a, Figure 3a, Figure 4a, Figure 5a and Figure 6a. These figures show the initial characteristics of the collaborators before collaboration by comparing the initial quality difference using the silhouette Index with the initial diversity.

As one can see, in the case of the easy data sets -small in size and with few attributes- we were able to generate random example that are representative of the full range of possible couples, see Figure 2a, Figure 3a and Figure 4a. However, for more complex data sets we were unable to cover the whole range of possible couples, see Figure 5a and Figure 6a. Specifically, the random generator could not find clustering results that have a similar quality index but a high diversity. Nevertheless, we believe that our results are still significant even for complex data sets.

We now move to the analysis of the influence of Diversity on the collaboration results. In Figure 2b, Figure 3b, Figure 4b, Figure 5b and Figure 6b, we show how the value of the Silhouette Index evolved during the collaboration depending on the initial diversity between the two clustering solutions before collaboration.

If we compare these results with the ones from two similar studies [22,26], we can say the following: While both studies use different quality indexes (purity which is very specific to SOM-based models and external indexes), they both conclude to the optimal diversity being around 0.5 which is contradictory with both our KKT study and our empirical results with points clouds. In our experiments do not show any performance spike around 50%. In our case, a higher diversity always induces a stronger potential for better results. By potential we mean that the best results were always achieved when the diversity was very high, but that a high diversity does not always imply good collaboration results. These divergences between our empirical results and the theoretical results from the previous section are discussed in more details in the conclusion section of this paper.

Finally, we now move to Figure 2c, Figure 3c, Figure 4c and Figure 5c and Figure 6c,d. In these figures, we show how the initial quality difference between two results before collaboration can influence the outcome of the collaboration. We conducted this study using two indexes: the Silhouette Index and the Davies-Bouldin Index.

We can see that for all data sets, most cases of negative and bad collaborations occur either when collaborating with solutions that have a similar or lower quality. When we cross these results with these on the influence of diversity, we can highlight that these negative collaboration areas are found when both the diversity and quality of the collaborators are low. We can clearly see that collaborators that have a very low comparative quality but a high diversity give better collaborative results than those that also have a lower quality but a low diversity. Our explanation for this result is that given our collaborative model, in the case of a very weak collaborator having a high diversity (i.e., an almost random solution vector), the collaborative term looses all its influence and only the local algorithm term matters. This therefore results an improvement despite the negative collaborator. However, in the case of a weak collaborator but a low diversity (weak local result), the collaborative term still has a lot of influence in the process and thus may lead to a deterioration of the results.

One possible weakness of this experiment is that it is not easy to determine whether the improvements on the results come mostly from the local term, the collaborative one, or both. A possible answer to this question could lie in Figure 5b,c, as well as Figure 6b,c, where we can see the the quality gain on the Silhouette index based on the initial diversity is a folded version of the quality gain depending on the initial quality difference. Based on these point clouds, and if we assume for a moment that all improvements with a weaker collaborator come only from the local term, the real influence of the collaborative term can be approximated on the diversity based diagrams and is still significant.

### 5.3. Regression Model from the Point Clouds

In this subsection, we show how to use point clouds similar to these of the previous section with the goal of extracting a model that could predict the outcome of a collaboration.

We Consider the following variables:

- $Q_{loc}$, the quality of the local clustering result before collaboration using the silhouette index. It is computed in the local feature space.
- $Q_{col}$, the quality of the collaborator clustering result before collaboration. It is computed using the Silhouette index in the local feature space of the algorithm receiving the collaboration.
- $\Delta Q=Q_{col}-Q_{loc}$, the quality difference between the receiving algorithm and the collaborating algorithm.
- *H*, the diversity with the collaborator before collaboration. We use our oriented entropy $H_{i,j}$ to get the potential information gain that the collaborator can provide.
- GQ, the quality improvement during the collaboration. It is the raw improvement on the silhouette index for the local algorithm. If the Silhouette index after collaboration is better than the one before, GQ>0. Otherwise, in case of a negative collaboration GQ≤0.

Please note that in this section, the notation *Q* refers to quality criteria (silhouette index, davies-bouldin index, etc.) and is unrelated with the *Q* from the objective functions described earlier.

For our point clouds, we re-used the same clouds than in the previous experiment with only two collaborators working together and we generated some extra points under the same conditions with the goal of having enough data to build a decent modem. In Table 2, we show the number of points used per data set to create this model.

We applied a Linear Regression algorithm to all point clouds with the goal of predicting GQ as described in Equation (25). We tried other regression models of higher orders, but the linear regression was ultimately the one that gave the best results.

(25) GQ=f(H,ΔQ)=a×H+b×ΔQ+c

We first tried to use $Q_{loc}$ and $Q_{col}$ as regression parameters, but the two always ended up with opposite regression factors which prompted us to use ΔQ as a regression factor instead. The resulting regression factors using ΔQ and *H* to predict the GQ the gain in quality are shown in Table 3.

Following these results, we ran again the linear regression algorithm on a global set grouping the simulations from all data sets. We obtained the results shown in Equations (26). The determination coefficient for GQ was evaluated to 89.80% with an absolute mean error of 0.0562. The mean errors and correlation coefficients using these equations on the individual data sets is shown in Table 4.

(26) GQ=0.3443×D+0.5195×ΔQ−0.0208

As one can see that the quality gain can be correctly approximated by a simple linear regression. Furthermore, this regression gives us a good idea of which parameters are the most important, with the quality being the most determining parameter, quickly followed by diversity.

From now on we will focus on the results for the prediction of the gain in quality GQ shown in Equation (26), Table 3 and Table 4.

First, we can see in Table 3 that diversity and quality can be used to predict the potential gain in quality after collaboration. The correlation coefficient after the linear regression is around 95%, and the average error on predicting the improvement of the silhouette index is bellow 5%. Moreover, we can clearly see that the regression factors for all the data sets are quite similar, with the notable exception of the Iris data set where the diversity and the quality difference have nearly the same weights. Finally, the regression factors were similar enough to run another linear regression on a merged version of all point clouds from all data sets. The resulting factors shown in Equation (26) gave similarly good performances on both the individual data sets, see Table 4 with determination coefficients still above 90% and absolute mean errors between 3% and 10%, as well as one the merged data point clouds with a determination coefficient around 90% and an average mean error around 6%. These facts tend to prove that it is possible to have a reliable idea of what the result of a collaboration will be in term of quality based on the characteristics of the different collaborators.

The factors themselves indicates that the quality difference has the most important impact followed by diversity. Suprisingly enough, the regression factors appears to be nearly identical for all datasets despite their very different nature and characteristics. They have a weight of approximately $\frac{1}{2}$ and $\frac{1}{3}$ respectively. Finally, all linear regression results featured a constant factor close to zero (between −0.08 and +0.05), the value of which may be dependent on the data set.

**Figure 7:**
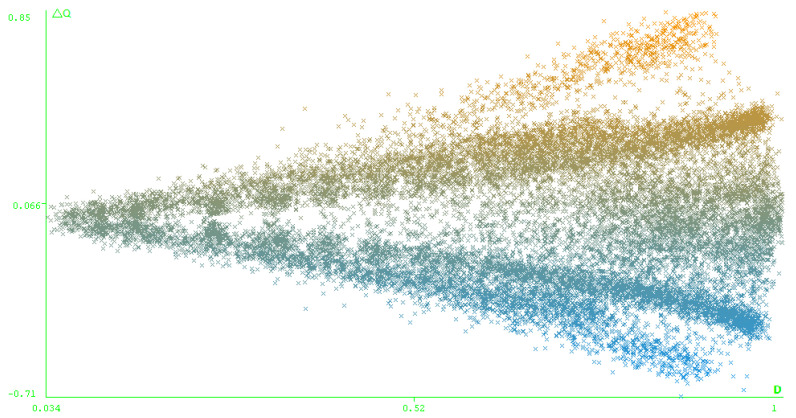
ΔQ=f(Diversity) for the merged data point clouds.

It is worth mentioning that the quality difference ΔQ and the diversity *D* that link two algorithms’ solutions are not completely independent from each others, see Figure 7. The potential to have a higher absolute quality difference between two clustering results grows when the diversity increases. It means that a low diversity will always mean a low difference in quality, but that the opposite is not true.

Based on the previous result, ideal collaboration link $\beta_{ji}$ given a known quality index to optimize and given entropy as a diversity measure would be defined as writen in Equation (27) below:

(27) $$\beta_{ji}=\max\left(0,\frac{1}{2}\left(Q_{i}-Q_{j}\right)+\frac{1}{3}H_{ji}-\lambda \right)$$

### 5.4. Weighting Proposition Based on the Regression Results

We now propose a short experiment where we analyze assess the proposed weights from Equation (27) and analyze the influence of the risk parameter λ. To do so, we use the same data sets than in the previous sections. The collaborative framework is as follows: we use 10 EM algorithms for the Gaussian mixture model working together through the EBCC algorithm. They are working on different features of the data sets. We repeat this procedure using different values of λ for the same initialization. For each value of λ, we assess the average quality gain during the collaboration. The quality index used in this experiment is the Silhouette index.

**Figure 8:**
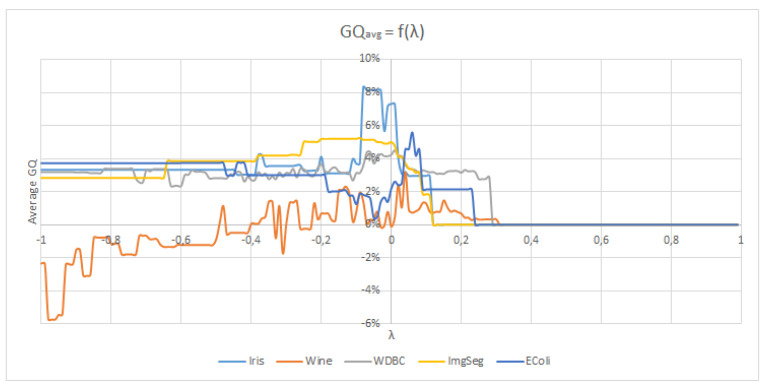
Evolution of the average improvement on the Silhouette Index ($GQ_{avg}$) depending on the chosen parameter λ.

In Figure 8, we show the results of this experiments for the Iris, Wine, WDBC, Image Segmentation and EColi data sets. As one can see, the average quality gain during the collaboration depending on the parameter risk λ follows a similar pattern for all data sets:

- For the lower values of λ (near −1) all collaborators are participating in the process with a relatively significant weight. In this case the collaboration results are poor (Wine data set) or average (the other data sets).
- When λ increases and gets closer to zero, only the best collaborators are given an important weight in the collaborative process and work together. For all the data sets, this range of values results in a sharp increase of the average quality gain during the collaboration.
- Finally, when λ becomes too high, there is no collaborator left that is considered good enough by the collaborative framework and the collaboration stops, hence the 0% average gain. For most data sets, this occurs around λ≈0.3.

From this experiment we can draw the conclusion that the quality prediction model introduced in Equation (26) is relatively accurate for several data sets and can be used to define the weights given to each algorithm during the collaboration. However, the performances of this model may vary greatly depending on the choice of the risk parameter λ, a parameter that has shown to be different depending on the data set. Finally, we can see that we the right values for λ, the results of Figure 8 outperforms the ones that we got from the same index in Table 1 with the KKT weighting system.

## 6. Conclusions and Perspectives

### 6.1. Discussion on the Influence of Quality and Diversity

In this subsection, we want to discuss the apparently contradictory results found in Section 4 and Section 5, as well as the results from earlier studies. In particular, we want to address the diverging results regarding the influence of diversity: In our KKT optimization study, we concluded that the best way to maximize the likelihood function of our algorithm is for each algorithm to favor solutions with which they have a low diversity. Yet, in the empirical study we show that while this is not the main factor, a higher diversity should be regarded as something desirable. Finally, other similar studies [22,23,26] reached the conclusion from their own experimental results that an average diversity between the solutions is the best option.

While these results may seem irreconcilable, one must look at them with pragmatism: our analysis for these divergences is that the 3 experiments are trying to determine optimal collaboration conditions for set ups that are very different.

In the case of the linear regression study in this paper compared with the studies Grozavu et al. and Rastin et al. [22,26], the goal was to find a setup that maximizes the collaborative outcome for an internal criterion in our case (the silhouette index), and external criteria in their case (the Adjusted Rand Index and node purity). What our own result says is that given a quality criterion *Q*, the best way to maximize this criterion during the collaboration is to collaborate with other algorithms that are as good as possible with this criterion, preferably much better than the local algorithm, thus favoring collaborators with a high quality and a high diversity. This result is very unsurprising and quite logical. In this regard our study, reaches the same conclusion as the other studies from the literature. The figures we obtained in this study are clouds for the influence of quality on quality result are even almost identical to the ones from the most recent study on SOM based collaborative clusetring [26].

However, when it comes to optimizing an external criterion such as the Adjusted Rand Index based on diversity and internal criterions, the strategy is quite different. Except for very easy data set, there is usually only a mild connection between internal and external criteria. Therefore choosing other collaborators based on the internal quality of their clusters makes little sense. Choosing collaborators with an average diversity is a safe compromise between collaborating with very different solutions that may be unstable and lead to negative collaboration, and collaborating with solutions that are too close and from which less will be learned toward improvement.

For the KKT approach, there was no criterion to optimize and nothing was known about the quality of the collaborators. Absent an internal or external quality criterion, the only thing that remains is stability [33]: if several algorithms come out with similar solutions, there is a good chance that the structures highlighted by these solutions are neither random nor some artifact. Therefore the mathematical result saying that in this set up it is best to collaborate with algorithms that have similar solutions makes sense because it tends to maximize the only criterion available: stability. Furthermore, for p>1, the results do not say to collaborate only with similar solutions, but merely to favor them.

This result highlights a conservative approach when trying to optimize a collaborative process with no specific criterion. As shown in the experimental results, this approach takes less risks thus leading to fewer cases of negative collaboration. But it also leads to weaker improvements because of the same lack of risk taking.

As a conclusion, we can say that the right method to weight the collaborators heavily depends on the criterion that one tries to maximize. While there may not be a universal answer, we want to highlight that the weighting method based on the KKT optimization is in our opinion the most convenient one: it relies on no specific quality index, it has an adjustable parameter *p* that allows to choose between conservatism and risk taking when weighting the collaborators, and it can be adapted to other collaborative frameworks. However, if you want to optimize a specific criterion (internal or not), it is best to choose collaborators that have good results with the said criterions.

We can therefore conclude that when there is a specific index to optimize, quality the most important factor, with diversity only providing room to explore new potentially good solutions. However, from our experimental results with the KKT optimization, we can also see that absent any specific quality index to optimize, diversity is the most important factor, and that low diversity collaborations should be favored as this increases the chances of having stable solutions. Furthermore, it may also be useful to regroup views with similar structures, and it may even help to detect noisy views.

### 6.2. Limitations of This Work

In this paper, we have proposed two methods to analyze the influence of diversity and quality in collaborative clustering. The first method that we proposed using KKT optimization was generic and applicable to many collaborative clustering methods, but the second method based on empirical results focused on the specific case of Entropy Based Collaborative clustering (EBCC) [13].

This raises a few questions and limits for our work: First, while our results seem to be coherent with the ones of other studies that used different algorithms, it is difficult to conclude that our findings are universal and will be true for any collaborative algorithm. Second, as highligthed in the work of Kuncheva et al. [16], the notion of diversity in ensemble learning can be assessed with many different indexes, and as such the choice to the global entropy may be a bias in our study. Finally, our study raises the question of the influence of partition stability as a potentially important factor, and this is a problem in the sense that this claim will prove very difficult to verify as clustering stability is by nature a difficult notion [32], and even more difficult to assess in practice.

### 6.3. Perspectives and Extensions to Other Algorithms

There are several perspectives to this work. The first one would be an extension of the empirical study to a higher number of algorithms. In this paper, we used only bilateral collaborations as they are easier to interpret and are less computationally intensive. However, despite the fact that higher degree collaborations may yield to results impossible to interpret, we are aware that bidirectional collaborations are not the majority of cases. As such, we may plan on exploring solutions to run similar analysis with a higher number of collaborators.

Another lead for future works would be to check if the empirical results can be verified with algorithms other than entropy based collaborative clustering. For instance, Kolmogorov based collaborative clustering [6] has slightly better performances and is compatible with the KKT study of this paper.

Finally, this paper opens new leads on studying the influence of the stability of the algorithms participating in collaborative and multi-view clustering processes, which is also an interesting research direction for future works.

## Figures and Tables

**Table 1:** Average Improvement after collaboration using the Karush Kuhn Tucker (KKT) weighting system in our collaborative framework, min and max improvement are between parenthesis.

Data Set	Silhouette Index	DB-Index
Wine	+1% $\left(\begin{array}{c} +3\%\\ -2\% \end{array}\right)$	+1% $\left(\begin{array}{c} +2\%\\ -1\% \end{array}\right)$
WDBC	+1% $\left(\begin{array}{c} +2\%\\ -1\% \end{array}\right)$	+1% $\left(\begin{array}{c} +3\%\\ +1\% \end{array}\right)$
Waveform	+4% $\left(\begin{array}{c} +15\%\\ +1\% \end{array}\right)$	+1% $\left(\begin{array}{c} +2\%\\ -1\% \end{array}\right)$
EColi	+9% $\left(\begin{array}{c} +34\%\\ -2\% \end{array}\right)$	+4% $\left(\begin{array}{c} +14\%\\ +1\% \end{array}\right)$
Image Segmentation	+4% $\left(\begin{array}{c} +12\%\\ 0\% \end{array}\right)$	+15% $\left(\begin{array}{c} +45\%\\ -3\% \end{array}\right)$

**Table 2:** Subsamples characteristics.

Data Set	Number of Collaborations
Iris	3000
Wine	3000
WDBC	3000
EColi	3000
ImgSeg	3000
VHR Strasbourg	500

**Table 3:** Regression factors for the prediction of GQ.

Data Set	*D*	ΔQ	Constant *c*	$R^{2}$	Abs. Mean Error
Iris	0.5612	0.5405	−0.0723	0.9683	0.0499
Wine	0.3262	0.4763	−0.0309	0.9718	0.0231
WDBC	0.3410	0.5337	−0.0032	0.9499	0.0302
Ecoli	0.3352	0.4518	0.0419	0.9510	0.0292
ImgSeg	0.2907	0.4438	0.0032	0.9221	0.0302
VHR Strasbourg	0.2456	0.4858	0.0212	0.9519	0.0100
Average	0.3363	0.4911	−0.0295	0.9513	0.03374

**Table 4:** Linear Regression Absolute Mean Errors and Correlation Coefficients.

Data Set	GQ Mean Error	$R^{2}$
Iris	0.0999	0.9547
Wine	0.0332	0.9717
WDBC	0.0526	0.9499
EColi	0.0361	0.9499
ImgSeg	0.0376	0.9223
VHR Strasbourg	0.0385	0.9090
